# Numerical Analysis of Curvature Effects on Acoustoelastic Surface Waves in Cylindrical Structures

**DOI:** 10.3390/s26041206

**Published:** 2026-02-12

**Authors:** Yongjiang Ma, Chunguang Xu, Changhong Chen, Shuangxu Yang

**Affiliations:** 1 School of Mechanical Engineering, Beijing Institute of Technology, Beijing 100081, China; 3120215244@bit.edu.cn (Y.M.); 3220225079@bit.edu.cn (C.C.); 3220235061@bit.edu.cn (S.Y.); 2 Key Laboratory of Fundamental Science for Advanced Machining, Beijing Institute of Technology, Beijing 100081, China

**Keywords:** acoustoelasticity, surface wave propagation, curvature effects, stress evaluation, finite element simulation

## Abstract

In this study, the influence of axial stress on surface wave propagation along cylindrical surfaces is investigated, with particular emphasis on quantifying curvature effects on acoustoelastic coefficients. The classical planar surface wave acoustoelastic formulation is first adopted as a reference. Three-dimensional transient finite element simulations are then performed to model surface wave excitation, propagation, and reception on aluminum cylinders with different radii and excitation frequencies. Stress-free simulations are used to extract surface wave velocities and reference time signals, while prestressed simulations provide stress-induced time delays, from which effective acoustoelastic coefficients are determined. The results indicate that both the surface wave velocity and the acoustoelastic coefficient exhibit clear dependencies on cylinder radius and excitation frequency. Curvature effects are especially pronounced at low frequencies, whereas at higher frequencies the coefficients corresponding to different radii tend to converge. These findings demonstrate that planar surface wave theory may lead to non-negligible errors when applied to cylindrical geometries and provide quantitative guidance for curvature-aware stress evaluation.

## 1. Introduction

Surface waves are widely used in nondestructive evaluation and material characterization due to their strong confinement near free surfaces and high sensitivity to surface and near-surface conditions [1,2,3]. In particular, surface wave-based techniques have been extensively employed for stress evaluation [4,5,6,7] through the acoustoelastic effect, where variations in wave velocity or travel time are related to the underlying stress state [8,9,10]. The majority of existing theoretical and experimental studies, however, are established within the framework of planar free surfaces.

In practical engineering applications, surface waves are frequently required to propagate along curved geometries, such as cylindrical rods, pipes, and shafts [11,12]. Despite this, it remains common practice to directly apply planar surface wave theory—including dispersion characteristics and acoustoelastic relations—to curved surfaces [7,13], implicitly assuming that surface curvature has a negligible influence on wave propagation. This assumption is generally valid only when the wavelength is sufficiently small compared with the radius of curvature. Outside this regime, surface curvature may significantly alter wavefields, phase velocity, and stress sensitivity, thereby introducing non-negligible errors in stress evaluation [14,15].

From a theoretical standpoint, a rigorous analysis of surface wave propagation on curved surfaces under prestress would require solving the acoustoelastic equation in curvilinear coordinates [16,17]. For cylindrical geometries, this entails expanding the governing equations in cylindrical coordinates and deriving the corresponding surface wave solutions, which is mathematically involved and does not generally lead to closed-form analytical expressions [18,19]. As a result, quantitative assessments of curvature effects on surface-wave acoustoelasticity remain limited.

To address this gap, the present study adopts a combined theoretical–numerical approach to quantify the influence of surface curvature on the acoustoelastic behavior of surface waves. The classical planar surface wave (Rayleigh wave) acoustoelastic formulation [20,21,22] is first taken as a reference model. Three-dimensional transient finite element simulations are then employed to investigate surface wave propagation on cylindrical structures with varying radii and excitation frequencies. By systematically analyzing stress-free and prestressed cases, curvature- and frequency-dependent corrections to the effective acoustoelastic coefficient are extracted.

The results demonstrate that surface curvature can substantially affect both the free surface wave velocity and its acoustoelastic response, particularly in the low-frequency regime. These findings indicate that direct application of planar surface wave theory to curved geometries may lead to significant inaccuracies. The framework proposed in this work provides a practical means to quantify and correct curvature-induced deviations, thereby improving the reliability of surface-wave-based stress evaluation in cylindrical structures.

## 2. Mathematical Framework

### 2.1. Formulation for Acoustoelastic Wave Propagation

The interaction between elastic wave propagation and the stress state of a solid medium is described by acoustoelastic theory [23,24,25]. When a material is subjected to a static deformation, its elastic response to superposed small-amplitude waves is modified, leading to stress-dependent wave velocities and dispersion characteristics. This coupling effect provides the theoretical basis for analyzing elastic wave propagation in pre-stressed solids.

Within the framework of incremental elasticity, the dynamic displacement field is treated as a small perturbation superposed on a statically deformed configuration. The static deformation is assumed to be sufficiently small such that terms higher than second order in strain may be neglected. Under these assumptions, the governing equations for the incremental motion remain linear with respect to the dynamic displacement, while the influence of the static stress state is incorporated through effective elastic moduli that depend on both second- and third-order elastic constants. As a result, elastic wave propagation in a pre-deformed solid is governed by a modified wave equation, commonly referred to as the acoustoelastic equation, which serves as the starting point for the subsequent analysis.

In principle, to rigorously analyze the influence of stress on surface-wave propagation along a cylindrical surface, the acoustoelastic equation should be formulated in a cylindrical coordinate system, fully accounting for curvature and axisymmetric stress distribution. However, such a formulation is extremely cumbersome and does not yield analytical solutions [16,18]. In the present study, we therefore adopt an approximate approach: the propagation of Rayleigh surface waves on a planar free surface is first analyzed, and the resulting acoustoelastic coefficients are subsequently corrected through numerical simulations to account for the cylindrical geometry and frequency-dependent effects.

In a Cartesian coordinate system associated with the initial configuration, the acoustoelastic equation takes the form of a second-order partial differential equation [26]:

(1) $$\frac{\partial }{\partial X_{J}}\left[\left(C_{IJKL}+\delta_{IK}t_{JL}^{i}\right)\frac{\partial u_{K}}{\partial X_{L}}\right]=\rho^{i}\frac{\partial^{2}u_{I}}{\partial t^{2}}$$

Here, $u_{I}$ represents the displacement components, $\rho^{i}$ is the mass density of the medium in the initial state, $\delta_{IK}$ is the Kronecker delta, and $t_{JL}^{i}$ is the initial Cauchy stress tensor. Equation (1) embodies the combined influence of material elasticity and initial stress on elastic wave propagation.

The effective stiffness tensor $C_{IJKL}$ incorporates contributions from second- and third-order elastic constants as well as the gradient of the initial displacement:

(2) $$\begin{array}{l} C_{IJKL}=c_{IJKL}\left(1-e_{NN}^{i}\right)+c_{IJKLMN}\frac{\partial u_{N}^{i}}{\partial X_{M}}\\ \;\;\;\;+c_{MJKL}\frac{\partial u_{I}^{i}}{\partial X_{M}}+c_{IMKL}\frac{\partial u_{J}^{i}}{\partial X_{M}}+c_{IJML}\frac{\partial u_{K}^{i}}{\partial X_{M}}+c_{IJKM}\frac{\partial u_{L}^{i}}{\partial X_{M}} \end{array}$$

Here, $c_{IJKL}$ denotes the fourth-order tensor whose components are second-order elastic constants, $c_{IJKLMN}$ denotes the six-order tensor whose components are third-order elastic constants, and $e_{NN}^{i}$ denotes the trace of the initial strain tensor.

### 2.2. Analysis of Rayleigh Wave Propagation

Consider the propagation of a Rayleigh surface wave along the free surface of a homogeneous elastic half-space subjected to a uniform static deformation. Under this assumption, Equation (1) simplifies to

(3) $$A_{IJKL}\frac{\partial^{2}u_{K}}{\partial X_{J}\partial X_{L}}=\rho^{i}\frac{\partial^{2}u_{I}}{\partial t^{2}}$$

where the effective elastic tensor is defined as:

(4) $$A_{IJKL}=C_{IJKL}+\delta_{IK}t_{JL}^{i}$$

The tensor $A_{IJKL}$ thus characterizes the effective elastic response of the pre-stressed medium through which elastic waves propagate.

**Figure 1:**
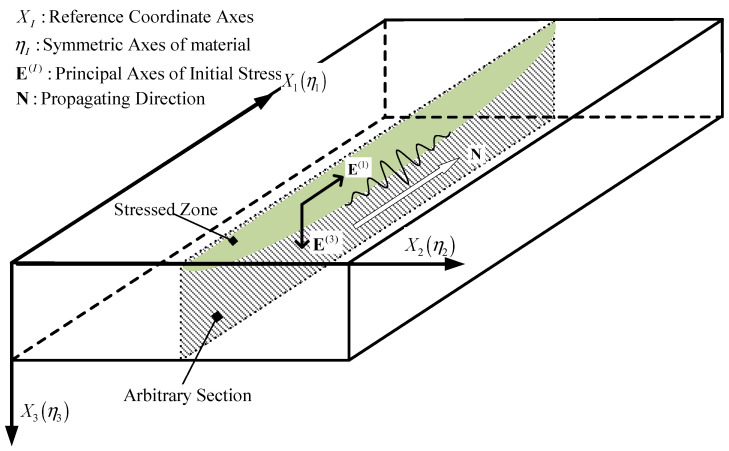
Alignment of principal strain axes with material symmetry directions.

The principal axes of strain $E^{\left(I\right)}$ are assumed to coincide with the material symmetry directions $\eta_{I}$, and the material coordinates are adopted as the reference coordinates $X_{I}$. The medium is assumed to occupy the region $X_{3}\geq 0$, and the wave propagates along the $X_{1}$ direction, as illustrated in Figure 1.

Owing to the symmetry conditions imposed above, motion polarized in the sagittal plane $\left(X_{1},X_{3}\right)$ decouples from the shear horizontal (SH) mode, and the displacement component $u_{2}$ associated with the SH mode may therefore be neglected [20]. Consequently, the analysis reduces to a two-dimensional problem involving only the in-plane displacement components associated with Rayleigh wave propagation. The incremental displacement field is thus assumed in harmonic form along the propagation direction, with amplitudes depending solely on depth [13,21,27]:

(5) $$u_{I}=f_{I}\left(x_{3}\right)\exp\left[iK\left(x_{1}-V_{R}t\right)\right]\;\left(I=1,3\right)$$

where $u_{2}=0$, $f_{I}$ denote the depth-dependent amplitudes, and $V_{R}$ is the Rayleigh wave velocity in the presence of stress.

Substitution of Equation (5) into the incremental equations of motion Equation (3) yields a coupled system of ordinary differential equations governing the depth-dependent amplitudes $f_{I}$. By introducing a scalar potential function, this coupled system can be reduced to a single fourth-order differential equation.

The free surface is assumed to be traction-free in both the static and incremental states. Imposition of the incremental traction-free boundary conditions leads to a homogeneous linear system for the modal amplitudes. The existence of non-trivial solutions requires the vanishing of the determinant of this system, which yields the Rayleigh-wave secular equation for a uniformly pre-strained elastic medium. In the absence of static deformation, this equation reduces to the classical Rayleigh secular equation for an unstressed half-space.

Since the static deformation is assumed to be small, the Rayleigh wave phase velocity is expanded to first order in the principal static strain components. Substitution of this expansion into the secular equation and retention of linear strain terms yield a system of algebraic equations determining both the Rayleigh wave velocity in the unstressed state and its first-order sensitivity to the applied static strains. This procedure establishes a direct connection between the wave velocity and the underlying acoustoelastic response of the material.

The relative variation of the Rayleigh-wave velocity is subsequently expressed in terms of the static strain components and, by means of linear elastic relations, recast in terms of the corresponding static stresses. The resulting expression [27] takes the form of a linear acoustoelastic law:

$$\frac{\Delta V_{R}}{V_{R}^{0}}=A_{R12}^{\left(1\right)}{t^{i}}_{11}+A_{R12}^{\left(3\right)}{t^{i}}_{33}$$

where $V_{R}^{0}$ denotes the Rayleigh-wave velocity in the unstressed configuration, ${t^{i}}_{11}$ and ${t^{i}}_{33}$ are the in-plane static stress components, and $A_{R12}^{\left(1\right)}$ and $A_{R12}^{\left(3\right)}$ are the corresponding acoustoelastic coefficients determined by the second- and third-order elastic constants of the material. The above notations are taken from [27] directly. These coefficients quantify the sensitivity of Rayleigh wave propagation to the applied stress state and provide a theoretical basis for stress characterization in elastically pre-deformed solids.

For the present study, which focuses on a uniaxial stress state along the axial direction of a cylinder, the equation above can be simplified accordingly. Specifically, under the assumption that the only nonzero static stress component is $t_{11}^{i}=\sigma$, with $t_{33}^{i}=0$, the linear acoustoelastic relation reduces to:

(6) $$\frac{\Delta V_{R}}{V_{R}^{0}}=C_{R}\sigma$$

where $C_{R}\equiv A_{R12}^{\left(1\right)}$ is the effective acoustoelastic coefficient for the uniaxial stress configuration. This coefficient encapsulates the combined influence of the second- and third-order elastic constants in the direction of stress.

In the context of cylindrical specimens, a rigorous theoretical description of the acoustoelastic effect for surface waves propagating along a cylindrical surface under axial stress would require the formulation of the full acoustoelastic equation in cylindrical coordinates. As discussed above, such a formulation is analytically intractable and does not readily yield closed-form solutions. Therefore, in the present work, it is assumed that the linear acoustoelastic relation retains the same functional form as Equation (6), while the corresponding coefficient $C_{R}$ for the cylindrical surface case is no longer treated as an intrinsic material constant. Instead, it is expected to depend on the surface curvature and the characteristics of wave excitation.

This assumption is physically motivated by the fact that geometric curvature fundamentally alters the dispersion characteristics of surface waves. Compared with the planar case, curvature modifies the modal structure and penetration depth of the surface wave, leading to frequency- and radius-dependent dispersion behavior. Consequently, the effective wave velocity and stress sensitivity are influenced by both geometry and excitation conditions, rather than being governed solely by material properties.

Accordingly, the acoustoelastic coefficient $C_{R}$ is treated here as an effective parameter that depends explicitly on the cylinder radius and the incident wave frequency. Its quantitative dependence on these parameters is investigated through numerical simulations, which implicitly capture the curvature-induced dispersion effects and provide the necessary correction to the planar acoustoelastic theory for surface-wave propagation on cylindrical surfaces.

### 2.3. Equivalent Acoustoelastic Coefficients for Wave Modeling

For comparison, consider the governing equation for elastic wave propagation in a homogeneous, linear elastic medium without initial stress [28]:

(7) $$c_{IJKL}\frac{\partial^{2}u_{K}}{\partial X_{J}\partial X_{L}}=\rho^{i}\frac{\partial^{2}u_{I}}{\partial t^{2}}$$

where $c_{IJKL}$ denotes the same fourth-order tensor composed of second-order elastic constants as in Equation (2).

By contrasting Equation (7) with the acoustoelastic wave equation Equation (3) derived in Section 2.2, it is evident that the influence of initial stress on wave propagation can be formally interpreted as a modification of the elastic constants through Equations (2) and (4).

In other words, the effect of initial stress enters the wave equation through the acoustoelastic tensor $A_{IJKL}$, which incorporates contributions from both second- and third-order elastic constants as well as the initial strain and stress fields. This observation motivates the introduction of an equivalent elastic stiffness tensor, by which the acoustoelastic effect may be embedded into conventional elastic wave models.

According to [29,30], when the material exhibits orthotropic or higher symmetry, the acoustoelastic tensor $A_{IJKL}$ may be replaced by an equivalent stiffness tensor ${\tilde{A}}_{IJKL}$ possessing orthotropic symmetry, such that

$${\tilde{A}}_{IJKL}={\tilde{A}}_{JIKL}={\tilde{A}}_{KLIJ}$$

This equivalence allows the acoustoelastic wave equation to be cast into the same mathematical form as that governing wave propagation in an orthotropic elastic medium.

Consequently, to incorporate acoustoelastic effects into numerical wave simulations, it is necessary to examine elastic wave propagation in orthotropic materials and to determine the corresponding equivalent elastic coefficients.

An orthotropic solid is characterized by three mutually orthogonal planes of material symmetry, whose intersections define the three principal material axes. In such materials, there are nine independent second-order elastic constants and twenty independent third-order elastic constants [31,32]. Using Voigt notation, with the index mapping

11→1, 22→2, 33→3, 32→4, 13→5, 21→6

the second-order elastic constants matrix $c_{pq}\left(p,q=1,2,\ldots ,6\right)$ takes the form

(8) $$c=\left[\begin{array}{cccccc} c_{11} & c_{12} & c_{13} & & & \\ c_{21} & c_{22} & c_{23} & & & \\ c_{31} & c_{32} & c_{33} & & & \\ & & & c_{44} & & \\ & & & & c_{55} & \\ & & & & & c_{66} \end{array}\right]$$

Similarly, the third-order elastic constants $c_{pqr}\left(p,q,r=1,2,\ldots ,6\right)$ possess twenty independent components under orthotropic symmetry, which may be listed as [26,33]:

$$\begin{array}{l} c_{111},\;c_{222},\;c_{333}\;\\ c_{144},\;c_{255},\;c_{336}\\ c_{112},\;c_{223},\;c_{133},\;c_{113},\;c_{112},\;c_{233}\\ c_{155},\;c_{244},\;c_{344},\;c_{166},\;c_{266},\;c_{355},\\ c_{123},\;c_{456}. \end{array}$$

In the present work, the material is assumed to be isotropic in its natural (unstressed) state, which leads to a further simplification of the elastic constants. For isotropic materials, the fourth-order tensor $c_{ijkl}$ is expressed in terms of the Lamé parameters λ and μ as

(9) $$c_{ijkl}=\lambda \delta_{ij}\delta_{kl}+\mu \left(\delta_{ik}\delta_{jl}+\delta_{il}\delta_{jk}\right)$$

The third-order elastic response of an isotropic solid is characterized by three independent Murnaghan constants l, m and n. The corresponding six-order tensor $c_{ijklmn}$ is given by [31]

(10) $$\begin{array}{l} c_{ijklmn}=l\delta_{ij}\delta_{kl}\delta_{mn}\\ +m\left(\delta_{ij}\delta_{km}\delta_{ln}+\delta_{ij}\delta_{kn}\delta_{lm}+\delta_{kl}\delta_{im}\delta_{jn}+\delta_{kl}\delta_{in}\delta_{jm}+\delta_{mn}\delta_{ik}\delta_{jl}+\delta_{mn}\delta_{il}\delta_{jk}\right)\\ +n\left(\delta_{ik}\delta_{jl}\delta_{mn}+\delta_{il}\delta_{jk}\delta_{mn}+\delta_{im}\delta_{jn}\delta_{kl}+\delta_{in}\delta_{jm}\delta_{kl}+\delta_{km}\delta_{ln}\delta_{ij}+\delta_{kn}\delta_{lm}\delta_{ij}\right) \end{array}$$

In Voigt notation, the third-order elastic constants $c_{pqr}$ of an isotropic material are related to the Murnaghan constants by

(11) $$\left\{\begin{array}{l} c_{111}=c_{222}=c_{333}=2l+4m\\ c_{144}=c_{225}=c_{366}=m-\frac{n}{2}\\ c_{112}=c_{223}=c_{133}=c_{113}=c_{122}=c_{233}=2l\\ c_{155}=c_{244}=c_{344}=c_{166}=c_{266}=c_{355}=m\\ c_{123}=2l-2m+n\\ c_{456}=\frac{n}{4} \end{array}\right.$$

Since the acoustoelastic wave equation can always be formulated in the material coordinate system, and the effective material symmetry considered here arises solely from the presence of initial stress, the material principal axes are taken to coincide with the principal stress axes. Under this assumption, the constitutive response induced by the initial stress preserves orthotropic symmetry with respect to the stress principal directions.

Furthermore, if the propagation direction of the elastic wave is aligned with one of the principal stress axes, all deviatoric stress components vanish, and the resulting acoustoelastic response is governed solely by the normal stress components.

Based on Equations (3) and (4), together with the intrinsic symmetry properties of the elastic stiffness tensors, the nonzero components of the acoustoelastic tensor $A_{IJKL}$ can be written as

(12) $$\left\{\begin{array}{l} A_{1111}=c_{11}+4c_{11}e_{11}^{i}+c_{111}e_{11}^{i}+c_{112}e_{22}^{i}+c_{113}e_{33}^{i}+t_{11}^{i}\\ A_{2222}=c_{22}+4c_{22}e_{22}^{i}+c_{122}e_{11}^{i}+c_{222}e_{22}^{i}+c_{223}e_{33}^{i}+t_{22}^{i}\\ A_{3333}=c_{33}+4c_{33}e_{33}^{i}+c_{133}e_{11}^{i}+c_{233}e_{22}^{i}+c_{333}e_{33}^{i}+t_{33}^{i}\\ A_{1122}=A_{2211}=c_{12}+2c_{12}\left(e_{11}^{i}+e_{22}^{i}\right)+c_{112}e_{11}^{i}+c_{122}e_{22}^{i}+c_{123}e_{33}^{i}\\ A_{2233}=A_{3322}=c_{23}+2c_{23}\left(e_{22}^{i}+e_{33}^{i}\right)+c_{123}e_{11}^{i}+c_{223}e_{22}^{i}+c_{233}e_{33}^{i}\\ A_{3311}=A_{1133}=c_{31}+2c_{31}\left(e_{11}^{i}+e_{33}^{i}\right)+c_{113}e_{11}^{i}+c_{123}e_{22}^{i}+c_{133}e_{33}^{i}\\ A_{2332}=A_{2332}=c_{44}+2c_{44}\left(e_{22}^{i}+e_{33}^{i}\right)+c_{144}e_{11}^{i}+c_{244}e_{22}^{i}+c_{344}e_{33}^{i}\\ A_{3113}=A_{1331}=c_{55}+2c_{55}\left(e_{11}^{i}+e_{33}^{i}\right)+c_{155}e_{11}^{i}+c_{255}e_{22}^{i}+c_{355}e_{33}^{i}\\ A_{1221}=A_{2112}=c_{66}+2c_{66}\left(e_{11}+e_{22}\right)+c_{166}e_{11}^{i}+c_{266}e_{22}^{i}+c_{366}e_{33}^{i}\\ A_{2323}=A_{2332}+t_{33}^{i},\;A_{3232}=A_{2332}+t_{22}^{i}\\ A_{3131}=A_{3113}+t_{11}^{i},\;A_{1313}=A_{3113}+t_{33}^{i}\\ A_{1212}=A_{1221}+t_{22}^{i},\;A_{2121}=A_{1221}+t_{11}^{i} \end{array}\right.$$

Here, the initial infinitesimal strain tensor is defined as

$$e_{IJ}^{i}=\frac{1}{2}\left(\frac{\partial u_{I}}{\partial X_{J}}+\frac{\partial u_{J}}{\partial X_{I}}\right)$$

Throughout this study, the initial stress state is idealized as a spatially uniform uniaxial stress field acting along the cylinder axis, with secondary stresses arising from boundary constraints assumed to be negligible. For this initial stress state, the acoustoelastic tensor can be further simplified. Under linear elasticity, the principal strains may be expressed in terms of the applied uniaxial stress σ and the Lamé parameters as

(13) $$\left\{\begin{array}{l} e_{11}^{i}=-\frac{\lambda \sigma }{2\mu \left(3\lambda +2\mu \right)}\\ e_{22}^{i}=-\frac{\lambda \sigma }{2\mu \left(3\lambda +2\mu \right)}\\ e_{33}^{i}=\frac{\left(\lambda +\mu \right)\sigma }{\mu \left(3\lambda +2\mu \right)} \end{array}\right.$$

Substitution of Equation (13) into Equation (12) yields the following expressions for the effective stiffness coefficients:

(14) $$\begin{array}{l} \left\{\begin{array}{l} A_{1111}=\lambda +2\mu +\frac{4\lambda^{2}+15\lambda \mu +4m\left(\lambda +\mu \right)+2\mu \left(l+5\mu \right)}{A_{0}}\sigma \\ A_{2222}=A_{3333}=\lambda +2\mu -\frac{2\left(\lambda^{2}+m\lambda +2\lambda \mu -l\mu \right)}{A_{0}}\sigma \\ A_{1122}=A_{1133}=\lambda +\frac{2\lambda^{2}+\left(2m-n+4\mu \right)\lambda +4l\mu }{2A_{0}}\sigma \\ A_{2233}=\lambda -\frac{2\lambda^{2}+\left(2m-n\right)\lambda -\left(2l-2m+n\right)\mu }{A_{0}}\sigma \\ A_{1221}=A_{3113}=\mu +\frac{8\mu^{2}+4\left(m+\lambda \right)\mu +n\lambda }{4A_{0}}\sigma \\ A_{2332}=\mu +\frac{\left(2m-4\lambda -n\right)\mu -n\lambda }{2A_{0}}\sigma \end{array}\right.\\ A_{2323}=A_{2332}=\;A_{3232}\\ A_{3131}=A_{3113}+t_{11}^{i},\;A_{1313}=A_{3113}\\ A_{1212}=A_{1221},\;A_{2121}=A_{1221}+t_{11}^{i} \end{array}$$

where $A_{0}\equiv \mu (3\lambda +2\mu )$.

Although the resulting tensor $A_{IJKL}$ does not strictly satisfy orthotropic symmetry, since

$$A_{3131}\neq A_{1313},A_{2121}\neq A_{1212}$$

it may be transformed into an equivalent orthotropic tensor following the procedure proposed in [34]. Specifically, the equivalent stiffness tensor ${\tilde{A}}_{IJKL}$ is defined as

(15) $$\left\{\begin{array}{l} {\tilde{A}}_{3131}={\tilde{A}}_{1313}=\frac{A_{3131}+A_{1313}}{2}\\ {\tilde{A}}_{2121}={\tilde{A}}_{1212}=\frac{A_{2121}+A_{1212}}{2}\\ {\tilde{A}}_{IJKL}=A_{IJKL},\;\mathrm{otherwise} \end{array}\right.$$

With this definition, the equivalent stiffness tensor ${\tilde{A}}_{IJKL}$ satisfies orthotropic symmetry and can be directly employed as a set of effective material parameters in conventional elastic wave propagation models. Consequently, the influence of initial stress on wave propagation may be incorporated into numerical simulations by replacing the elastic stiffness tensor $c_{IJKL}$ in the standard wave equation with ${\tilde{A}}_{IJKL}$.

It is noted that the symmetrization procedure in Equation (15) constitutes a numerical approximation introduced to facilitate orthotropic material modeling; its influence is expected to be secondary relative to the dominant curvature- and dispersion-induced effects examined in this study.

## 3. Simulation of Cylindrical Surface Wave Propagation by FEM

To quantify the dependence of the effective acoustoelastic coefficient $C_{R}$ on geometrical curvature and excitation frequency, a series of finite-element simulations were carried out using an explicit dynamic formulation. The simulations were designed to reproduce the excitation, propagation, and reception of surface waves traveling along the cylindrical surface of an axially stressed solid.

Cylindrical specimens with radii of 10, 12, 15, 18, and 20 mm were considered. All models were assigned the material properties of aluminum alloy 6061, which can be derived from those listed in Table 1. Surface waves were excited at center frequencies of 100 kHz, 200 kHz, 500 kHz, 800 kHz, and 1 MHz, covering a broad range of wavelength-to-radius ratios. For each radius–frequency combination, transient wave propagation along the cylindrical surface was simulated, and surface wave signals were recorded at prescribed receiver locations.

**Figure 2:**
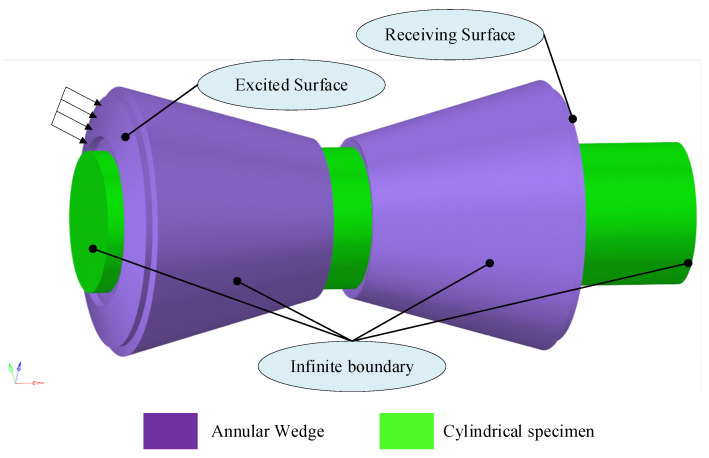
Setup of Simulation Model.

To generate surface waves in the numerical model, an equivalent excitation scheme was employed to represent the effect of a piezoelectric transducer coupled with an annular PMMA wedge, whose material properties are listed in Table 1. Instead of explicitly modeling the electromechanical behavior of the piezoelectric element, a prescribed time-dependent mechanical loading in the form of a four-cycle Hanning-windowed tone burst was applied at the wedge boundary. This excitation produces an obliquely incident bulk wave within the annular PMMA wedge, as illustrated in Figure 2. By appropriately selecting the wedge angle, the incident bulk wave undergoes mode conversion at the wedge–cylinder interface, thereby generating a surface wave that propagates along the cylindrical surface. This modeling strategy effectively captures the essential physics of wedge-based surface wave excitation while significantly reducing computational cost.

The simulated surface wave signals were subsequently analyzed to extract phase velocities under different axial stress levels. By comparing stress-dependent velocity variations with the unstressed reference state, the effective acoustoelastic coefficient $C_{R}$ was identified for each radius and excitation frequency. These results provide numerical corrections to the planar acoustoelastic theory and establish the functional dependence of $C_{R}$ on both cylinder radius and excitation frequency.

### 3.1. Numerical Model and Simulation Setup

All the cylindrical specimens had the same total axial length of 90 mm, which was sufficient to ensure that the surface wave propagated over a stable distance before reaching the receiving region. The cylindrical specimen was discretized using a mesh composed entirely of cubic hexahedral (brick) elements, and the annular PMMA wedge was meshed predominantly with hexahedral elements, with a characteristic element size of 0.2 mm across the computational domain. This mesh resolution was selected to adequately resolve the shortest wavelength associated with the highest excitation frequency considered in this study.

An explicit time-integration scheme was employed. The time step was set to 10^−9^ s to satisfy the stability requirements of the transient dynamic analysis and to accurately capture the high-frequency wave motion. The total simulation time was 80 μs, which allowed for complete generation, propagation, and reception of the surface waves within the computational domain.

Thus, the simulations were performed using an explicit time-integration scheme, for which numerical stability is governed by a CFL-type condition. With a uniform hexahedral element size of $h_{\min}=0.2$ mm and the maximum dilatational wave speed in aluminum ($c_{L}\approx 6.3\times 10^{3}$ m/s), the critical stable time increment is on the order of $\Delta t_{\mathrm{crit}}\approx h_{\min}/c_{L}\sim 3.2\times 10^{-8}$ s (~32 ns). The adopted time increment Δt=1 ns is therefore at least one order of magnitude smaller than $\Delta t_{\mathrm{crit}}$, ensuring stable explicit integration with a conservative safety margin.

For spatial resolution, the shortest wavelength associated with the maximum excitation frequency $f_{\max}=1$ MHz is estimated using the Rayleigh-wave speed ($c_{R}\sim 2.9\times 10^{3}$ m/s), giving $\lambda_{R}\sim 2.9$ mm and approximately $\lambda_{R}/h_{\min}\approx 14–15$ elements per wavelength.

In time, $f_{\max}=1$ MHz corresponds to a minimum period of 1 μs, which is resolved by 1000 time steps with Δt=1 ns. These estimates indicate that both stability and spatiotemporal resolution requirements are comfortably satisfied for the present transient simulations.

To suppress spurious wave reflections from the axial boundaries of the cylinder and the corresponding boundaries of the annular wedge, infinite elements were applied at both end faces of the cylindrical specimen as well as on the adjacent axial surfaces of the wedge, as illustrated in Figure 2. This boundary treatment effectively absorbs outgoing waves and emulates an unbounded axial domain, thereby ensuring that the recorded surface-wave signals are not contaminated by end reflections.

### 3.2. Effects of Frequency and Radius on Surface Wave Propagation

**Figure 3:**
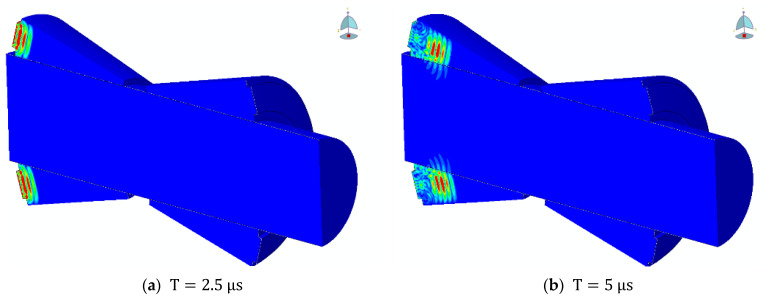

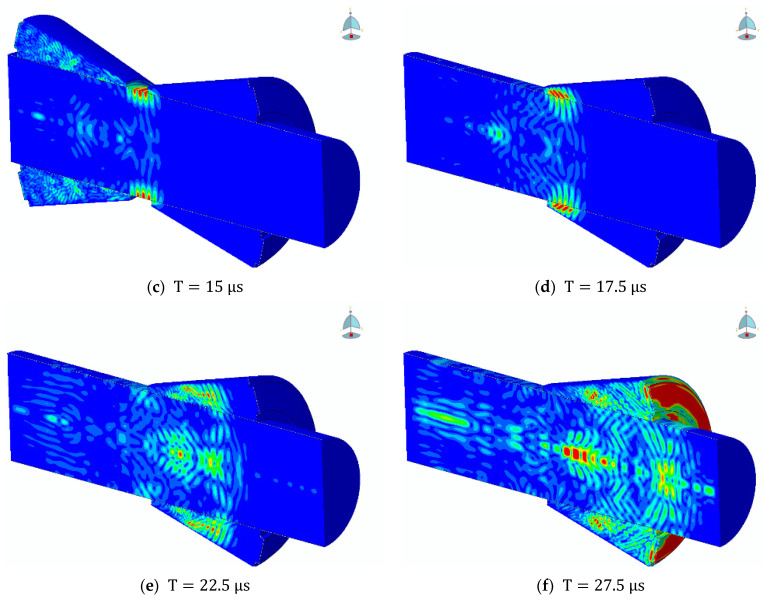
Transient evolution of surface-wave propagation.

Figure 3a–f present the time evolution of the displacement field obtained from the transient simulation for the case of a 1 MHz excitation applied to a cylindrical specimen with a radius of 10 mm. The displacement contours illustrate the complete process of surface wave excitation, propagation along the cylindrical surface, and subsequent reception.

At the initial stage (T = 2.5 to 5 μs), the excitation generated by the simulated piezoelectric transducer is transmitted into the specimen through the annular PMMA wedge, producing a localized displacement field near the excitation region. Owing to the refraction-induced phase velocity matching at the PMMA-6061 interface, a surface wave is efficiently generated on the cylindrical surface.

As time progresses (T = 5 to 15 μs), the surface wave propagates predominantly along the axial direction of the cylinder while remaining confined to a shallow region beneath the surface, exhibiting the characteristic exponential decay of displacement amplitude with depth. This behavior is consistent with the fundamental properties of Rayleigh-type surface waves.

At later times (T = 17.5 to 27.5 μs), the surface wave reaches the receiving location, where a clear and coherent displacement pattern can be observed. Throughout the propagation process, no significant wave reflections from the axial boundaries are visible, confirming the effectiveness of the infinite-element boundary treatment adopted at both ends of the cylinder.

These displacement contours demonstrate that the numerical model successfully captures the generation and propagation of surface waves on a cylindrical surface and provides a reliable basis for subsequent quantitative analysis of wave velocity and acoustoelastic effects.

**Figure 4:**
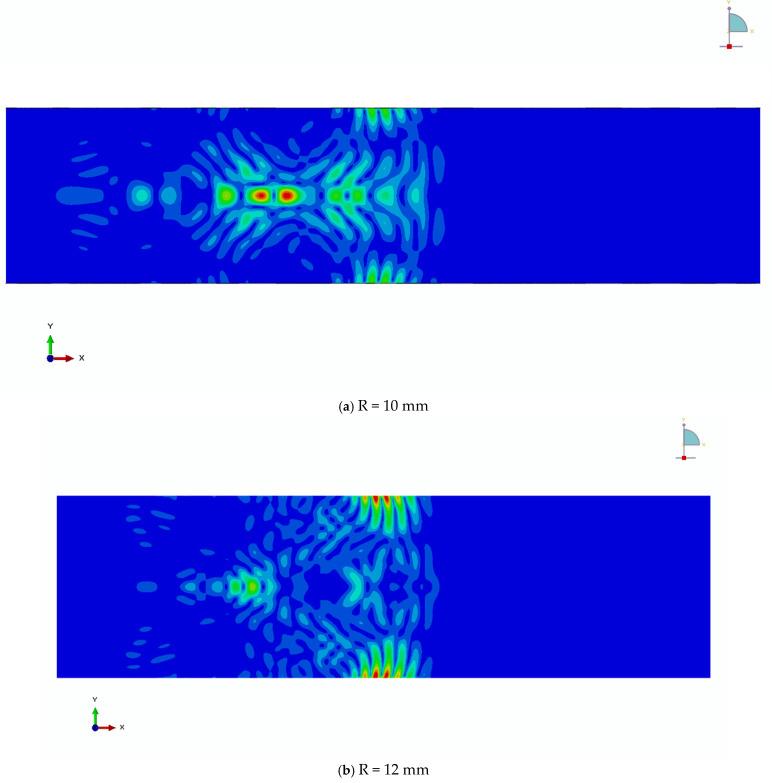

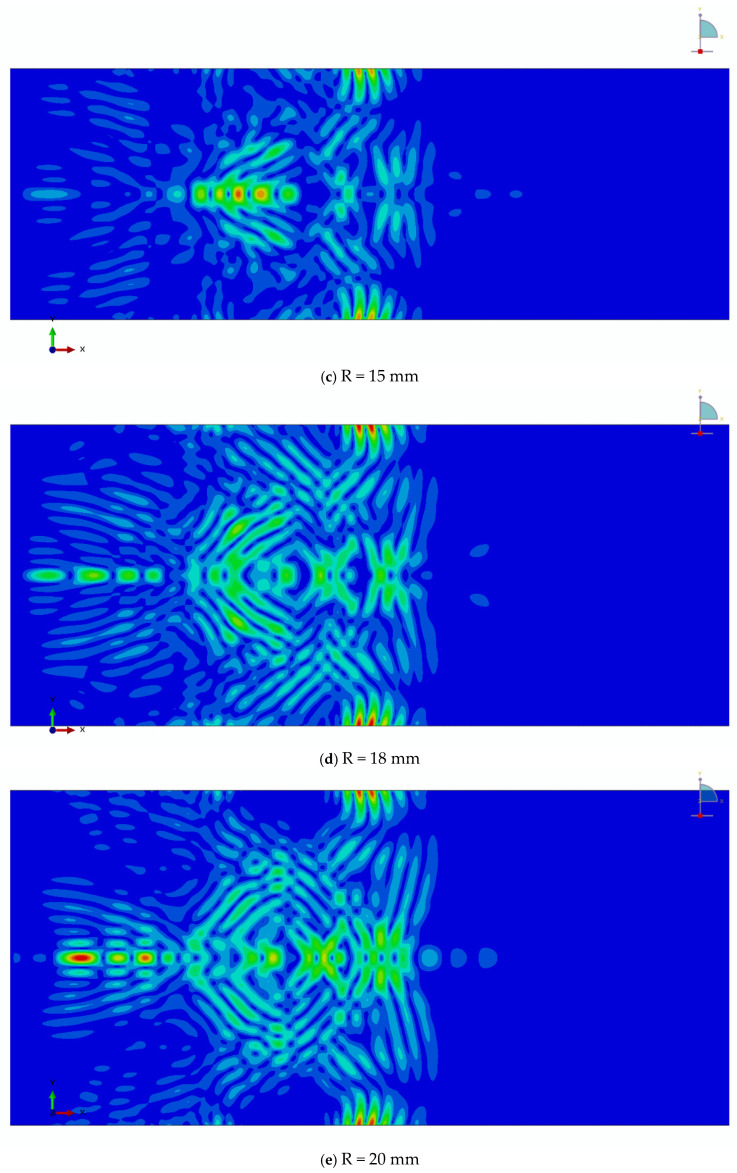
Displacement contours of fully developed surface waves at 1 MHz for cylindrical models with different radii, illustrating the influence of surface curvature on the wave field.

Figure 4a–e presents displacement contour snapshots of the surface wave at the fully developed propagation stage for models with different cylinder radii under a fixed excitation frequency of 1 MHz. All snapshots are taken at the same normalized propagation stage to ensure a consistent comparison among different geometrical configurations.

Although the excitation conditions and material properties are identical, the displacement fields exhibit noticeable differences as the cylinder radius varies. For larger radii, the surface-wave displacement pattern closely resembles that of a Rayleigh wave propagating on a planar surface, with the wavefront remaining well localized near the outer surface. As the radius decreases, curvature effects become increasingly pronounced, leading to a visible modification of the displacement distribution.

In particular, for smaller radii, the surface wave exhibits enhanced spatial spreading and a redistribution of displacement amplitude, indicating a stronger coupling between the surface wave and the cylindrical geometry. These observations suggest that the curvature of the propagation surface plays a non-negligible role in shaping the surface wave field, even in the absence of applied stress.

The differences observed in Figure 4 provide direct numerical evidence that the cylindrical geometry alters the surface wave characteristics relative to the planar case. This geometric dependence motivates the introduction of a radius-dependent acoustoelastic coefficient in the subsequent analysis.

It is worth noting that the curvature-induced modification of the surface wavefield observed in Figure 4a–e is qualitatively consistent with classical surface-wave theory for curved surfaces. According to Viktorov’s theory [35], surface curvature alters the distribution of particle motion and the effective penetration depth of surface waves, leading to deviations from planar Rayleigh wave behavior as the ratio of wavelength to curvature radius increases. In the present simulations, similar trends are observed: for smaller radii or lower excitation frequencies, the surface wave exhibits deeper penetration and a less localized wavefield, whereas for larger radii or higher frequencies the wavefield becomes increasingly confined near the surface, approaching the planar limit. While classical curved-surface theories do not readily provide explicit phase-velocity expressions applicable to the finite-radius and frequency-dependent configurations considered here, the numerical results presented in this study offer a practical means of quantifying these curvature effects.

**Figure 5:**
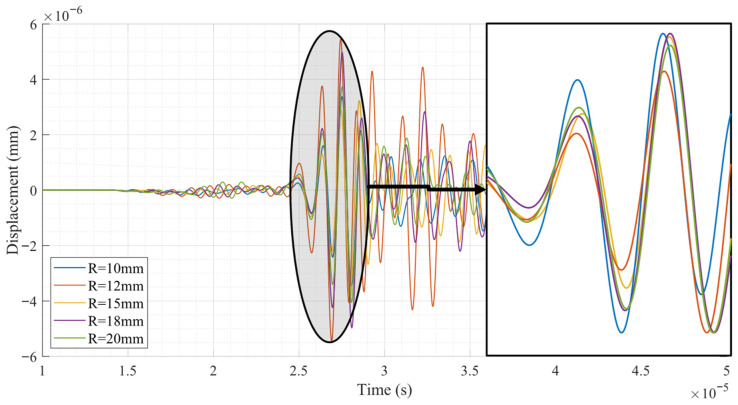
Surface-wave displacement signals for cylindrical models with different radii, illustrating radius-dependent variations in amplitude and phase.

Figure 5 shows the surface-wave displacement signals recorded at the receiving location for cylindrical models with different radii, within the time window from 10 μs to 50 μs. No discernible signal is observed prior to 10 μs, while beyond 50 μs the surface wave has already passed the receiver; therefore, this time interval captures the complete and relevant portion of the received surface wave response.

The recorded signals exhibit clear differences in both amplitude and phase as the cylinder radius varies. In particular, a systematic phase shift among the waveforms can be observed, indicating a radius-dependent variation in the surface-wave propagation velocity. As the cylinder radius decreases, the arrival time of the surface wave is progressively advanced, corresponding to an increase in the effective surface wave velocity. In fact, the surface-wave velocities listed in Table 2 are extracted directly from the arrival times of these leading wave packets at prescribed receiver locations.

This trend demonstrates that surface curvature has a measurable influence on surface-wave propagation characteristics, even under identical excitation frequency and material properties. The observed increase in wave velocity for smaller radii is attributed to the geometric constraint imposed by the curved surface, which modifies the stress-free boundary condition and the associated modal structure of the surface wave.

The differences in amplitude are mainly associated with geometric spreading and curvature-induced redistribution of the displacement field, as previously observed in the displacement contours. These time-domain results provide a direct basis for quantitative extraction of surface wave velocities and, subsequently, for evaluating the dependence of the acoustoelastic coefficient on cylinder radius and excitation frequency.

**Figure 6:**
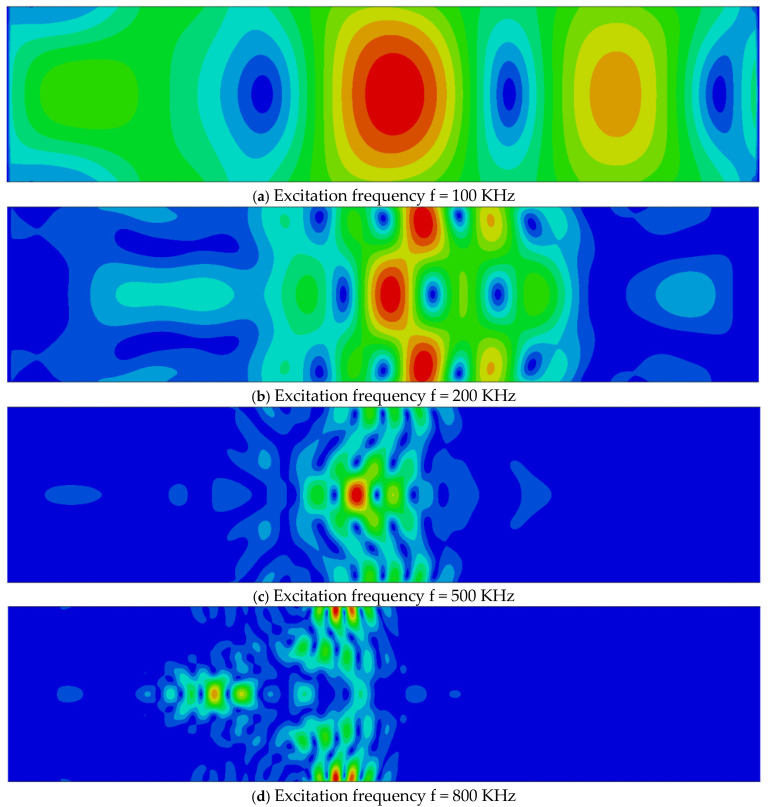
Displacement contours at the fully developed surface wave stage for the R10 cylindrical model under different excitation frequencies, illustrating the frequency-dependent surface-wave characteristics.

Figure 6a–d show displacement contour snapshots at the fully developed propagation stage for the R10 cylindrical model under excitation frequencies of 100, 200, 500, and 800 kHz, respectively. These results illustrate the pronounced influence of excitation frequency on the surface wave field.

At low excitation frequencies, the surface-wave condition approaches an extreme regime. In particular, at 100 kHz, the displacement field is no longer confined to a shallow surface region but instead occupies nearly the entire cross-section of the cylinder with radius R = 10 mm, indicating that the wave can no longer be regarded as a classical Rayleigh-type surface wave. At 200 kHz, although surface localization begins to emerge, the wavelength remains comparable to the cylinder diameter, and only a few wavelengths are accommodated across the cross-section.

As the excitation frequency increases to 500 kHz and above, the displacement field becomes increasingly localized near the cylindrical surface and exhibits the characteristic features of a well-developed surface wave. In this frequency range, the wave structure is qualitatively similar to that observed in the higher-frequency cases discussed previously, and the influence of surface curvature is less dominant in shaping the overall displacement pattern.

A comparison with the radius-dependent results presented earlier indicates that, within the investigated parameter range, the excitation frequency has a more significant impact on the surface wave characteristics than the cylinder radius. This observation highlights the importance of frequency selection in both numerical modeling and experimental measurements and suggests that frequency-dependent effects must be explicitly accounted for when evaluating the acoustoelastic response of cylindrical surface waves.

**Figure 7:**
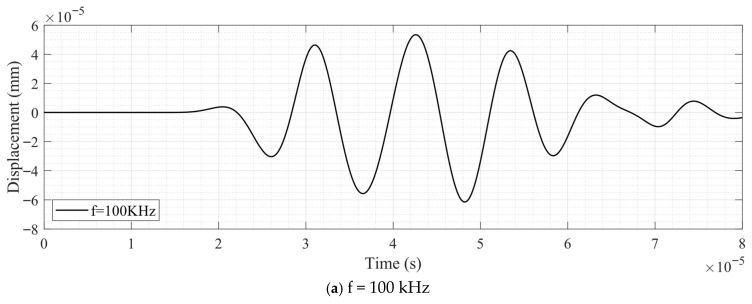

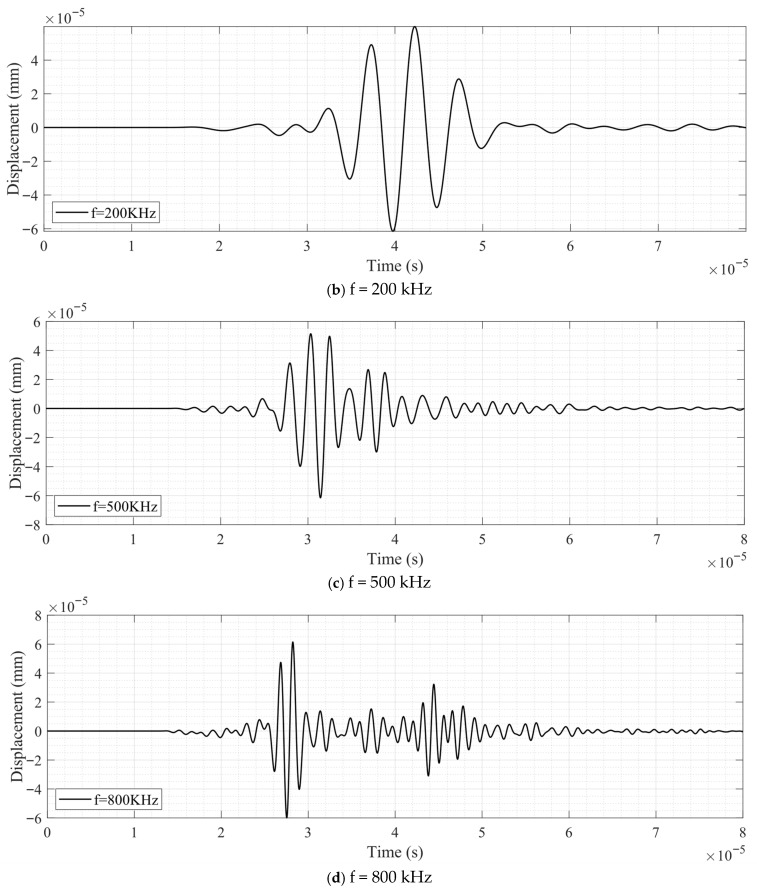
Received surface wave displacement signals for the R10 cylindrical model under excitation frequencies, shown over the complete time interval of 0 to 80 μs.

Figure 7a–d present the surface wave displacement signals recorded at the receiving location for the R10 cylindrical model under excitation frequencies of 100, 200, 500, and 800 kHz, respectively. Owing to the substantial differences in waveform characteristics across frequencies, the received signals are shown in four separate plots rather than being superimposed, as was done in Figure 5. For the same reason, the complete time history over the interval 0 to 80 μs is displayed for each frequency.

At lower excitation frequencies (100 and 200 kHz), the received surface-wave signals exhibit a clean and well-defined wave packet with a single dominant arrival. The waveform shape closely resembles that of the incident excitation, indicating minimal modal interference and stable propagation behavior. This observation is consistent with the displacement field distributions shown previously and suggests that the surface wave mode is well separated from other wave components in this frequency range.

At higher excitation frequencies (500 and 800 kHz), additional waveform components appear following the main surface wave arrival. These secondary features are attributed to weak interference from other wave modes and geometric effects associated with higher-frequency propagation on the curved surface. Nevertheless, the leading portion of the received signal remains well defined and exhibits a clear and repeatable phase evolution.

Since the present study focuses on surface wave velocity rather than full waveform reconstruction, only the initial cycles of the leading surface wave packet are used for velocity extraction. The presence of later time waveform disturbances therefore does not affect the accuracy or reliability of the velocity measurements employed in the subsequent acoustoelastic analysis.

A comparison of the arrival times across Figure 7a–d further indicates that the surface wave velocity increases as the excitation frequency decreases, consistent with the trends observed in the displacement contour plots. This frequency-dependent variation in wave velocity provides an important basis for the subsequent evaluation of the acoustoelastic coefficient.

As demonstrated above, the stress-free surface wave velocity $V_{R}$ is not a constant but depends on both the excitation frequency and the cylinder radius. For each frequency–radius combination, $V_{R}$ is extracted directly from the simulated received waveforms by measuring the arrival time difference of the maximum wave peak between two prescribed locations along the propagation path. Based on this procedure, a 5×5 matrix of stress-free surface wave velocities corresponding to the different excitation frequencies and cylinder radii is obtained and summarized in Table 2.

Since the surface-wave wavelength *λ* is determined by the phase velocity and the excitation frequency, the combined influence of frequency and cylinder radius on surface-wave behavior can be more physically characterized through the wavelength-to-radius ratio *λ*/*R*. This dimensionless parameter provides a direct measure of the relative importance of surface curvature effects, with larger values indicating stronger deviations from planar surface-wave behavior. Based on the stress-free surface wave velocities listed in Table 2, the corresponding *λ*/*R* ratios for all frequency–radius combinations are calculated and summarized in Table 3.

## 4. Analysis of the Simulated Acoustoelastic Effect

In Section 3, the numerical results for surface wave propagation in cylindrical models with varying geometrical parameters and excitation frequencies are presented, with particular emphasis on the received surface wave signals. These results correspond to the propagation of surface waves in the unstressed configuration and provide reference data for subsequent analysis.

To investigate the influence of prestress on surface wave propagation, the simulations were extended in this section to include prestressed configurations. Following the procedure described in Section 2.3, the effect of axial stress was incorporated by modifying the effective material parameters so as to account for acoustoelasticity. In this way, surface wave propagation under prestress was simulated without explicitly introducing static deformation into the geometric model.

For each numerical model, five prestress levels were considered, corresponding to axial stresses ranging from 40 to 200 MPa in increments of 40 MPa. For each prestress level, the surface wave excitation, propagation, and reception processes were simulated, and the corresponding displacement signals at the receiving location were recorded.

To quantify the stress-induced time delay, the earliest arriving surface-wave packet is first identified from the received waveform. One complete cycle of this leading wave packet is then extracted within a prescribed time window to minimize the influence of trailing waves and potential mode interference. The time delay between the stress-free and prestressed signals is evaluated using normalized cross-correlation applied to the extracted waveforms.

The transient simulations are performed with a temporal resolution of 1 ns. Prior to cross-correlation, the extracted waveforms are interpolated to an effective temporal resolution of 0.1 ns, enabling more accurate estimation of small time shifts. The resulting time delays are subsequently used to determine the effective surface-wave velocity variations and the corresponding acoustoelastic coefficients.

**Figure 8:**
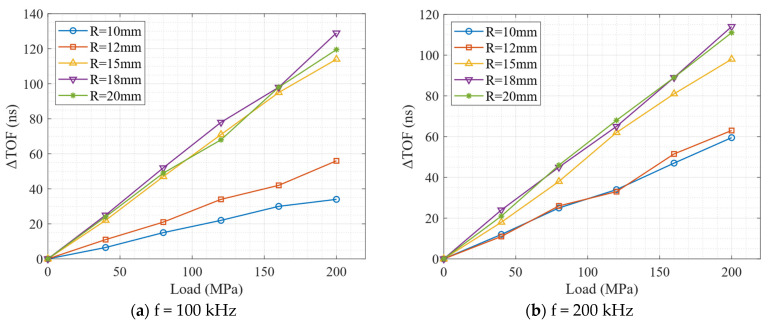

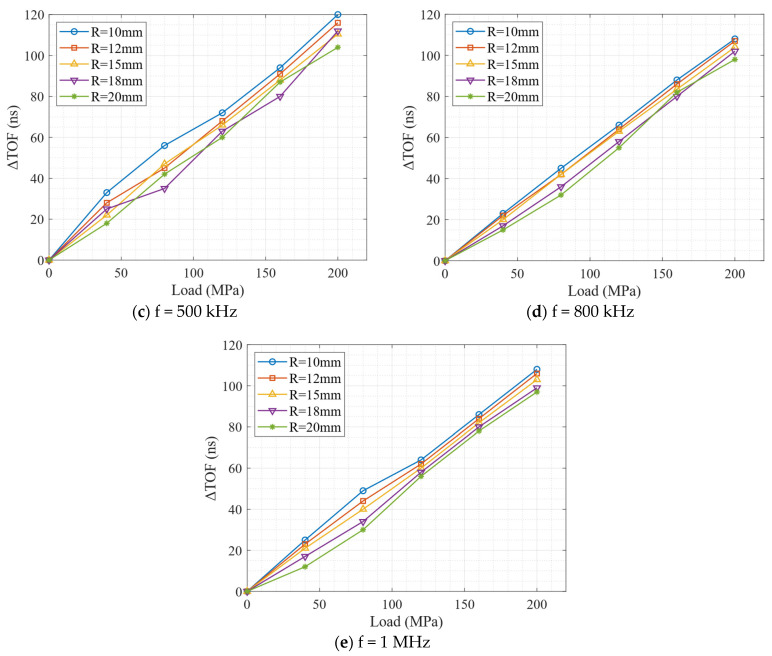
Stress-induced time delay of surface waves for different radii and excitation frequencies.

Figure 8a–e present the stress-induced time-delay (ΔTOF) variations of the received surface wave signals for the five excitation frequencies considered in this study. In each subfigure, five curves are shown, corresponding to cylindrical models with different radii. All curves pass through the origin, indicating that the ΔTOF vanishes in the absence of applied stress, as we assumed. Moreover, the relationships between ΔTOF and axial stress are approximately linear over the investigated stress range.

The slope of each curve characterizes the acoustoelastic effect and reflects the sensitivity of surface wave propagation to the applied axial stress. Distinct differences in slope can be observed for different excitation frequencies, indicating a clear frequency dependence of the acoustoelastic response. In addition, for a given excitation frequency, the slopes corresponding to different cylinder radii are not identical, demonstrating the influence of geometric curvature on the stress sensitivity of surface waves.

This radius dependence is particularly pronounced at low excitation frequencies (100 kHz and 200 kHz), where substantial differences in slope are observed between small- and large-radius models. In contrast, at higher frequencies, the slopes associated with different radii become much closer to one another and nearly collapse onto a single line. This behavior suggests that, within the investigated parameter range, the influence of excitation frequency on the acoustoelastic response is more significant than that of the cylinder radius.

**Figure 9:**
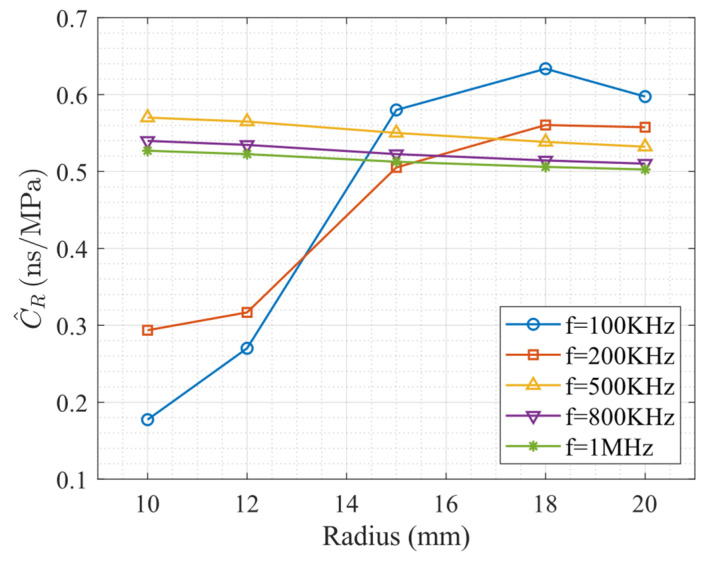
Acoustoelastic coefficient ${\hat{C}}_{R}$ as a function of cylinder radius for different excitation frequencies.

Figure 9 illustrates the variation of the acoustoelastic coefficient ${\hat{C}}_{R}$, obtained from the slopes of the linear fits in Figure 8, as a function of cylinder radius for five different excitation frequencies.

For each fixed excitation frequency, the acoustoelastic coefficient ${\hat{C}}_{R}$ exhibits a frequency-dependent sensitivity to the cylinder radius. At low frequencies (100 kHz and 200 kHz), ${\hat{C}}_{R}$ shows a pronounced dependence on radius. In particular, for the 100 kHz case, the coefficient increases rapidly as the radius grows from 10 mm to approximately 15–18 mm, followed by a slight saturation or decrease at larger radii. This non-monotonic behavior indicates that, under low-frequency excitation, the surface wave field is not strictly confined to the near-surface region and is therefore strongly influenced by the geometric curvature of the cylinder.

In contrast, at intermediate and high frequencies (500 kHz, 800 kHz, and 1 MHz), the dependence of ${\hat{C}}_{R}$ on radius becomes significantly weaker. For the 800 kHz and 1 MHz cases in particular, the coefficient remains nearly constant across the entire range of radii considered, with only a slight decreasing trend. This behavior suggests that, at higher frequencies, the surface wave becomes more localized near the surface, thereby reducing the influence of curvature on the acoustoelastic response.

When the radius is held constant, clear differences can be observed among the curves corresponding to different excitation frequencies. Overall, the low-frequency cases (100 kHz and 200 kHz) yield acoustoelastic coefficients that differ markedly from those obtained at higher frequencies, especially for smaller radii (10–12 mm), where ${\hat{C}}_{R}$ is substantially lower.

As the radius increases, the discrepancy between low- and high-frequency results gradually diminishes. For radii exceeding approximately 15 mm, the acoustoelastic coefficients obtained at low frequencies approach those of the higher-frequency cases. For excitation frequencies above 500 kHz, the curves cluster closely together, indicating that the frequency dependence of ${\hat{C}}_{R}$ becomes weak in the high-frequency regime.

These results demonstrate that the acoustoelastic coefficient of cylindrical surface waves is not a single material constant, but rather an effective parameter jointly governed by excitation frequency and geometric scale. Within the range investigated, the influence of excitation frequency is more pronounced than that of cylinder radius. In particular, geometric curvature plays a dominant role in modulating the acoustoelastic response at low frequencies, whereas at higher frequencies the coefficient tends to converge toward behavior characteristic of surface waves propagating on a planar surface.

Figure 9 summarizes the acoustoelastic coefficients obtained directly from the slopes of the Stress–ΔTOF curves shown in Figure 8. These coefficients characterize the sensitivity of the surface wave time delay to axial stress but do not yet account for variations in the stress-free surface wave velocity. Based on the above analysis, the acoustoelastic coefficient extracted in this section is defined through the relation

(16) $$\sigma ={\hat{C}}_{R}\Delta \mathrm{TOF}$$

This definition differs from the acoustoelastic coefficient $C_{R}$ introduced in Equation (6). The two coefficients are, however, closely related.

Using the apparent relations

(17) $$\frac{\Delta \mathrm{TOF}}{T}=-\frac{\Delta V_{R}}{V_{R}^{0}};L_{\mathrm{eff}}=V_{R}^{0}T$$

where $L_{\mathrm{eff}}$ is the effective acoustic path length and T is the total propagating time, one obtains

(18) $$C_{R}=-\frac{V_{R}^{0}}{L_{\mathrm{eff}}}{\hat{C}}_{R}$$

For all numerical models considered in this study, the effective propagation length may be taken as *L*_eff_ = 60 mm.

By substituting the velocity values from Table 1 into the above relation Equation (18), the corresponding values of $C_{R}$ are obtained.

For clarity, a representative example is provided for the case R=10 mm and f=100 kHz. From the numerical results shown in Figure 8a, the slope of the stress–time delay curve yields an effective coefficient of ${\hat{C}}_{R}=0.177$ ns/MPa, obtained through linear regression and interpolation of the simulated data. According to Equation (18), the corresponding normalized acoustoelastic coefficient $C_{R}$ is calculated as

$$C_{R}=-0.177\;\mathrm{ns}/\mathrm{MPa}\times \frac{4909.39\;m/s}{60\;\mathrm{mm}}=-14.51\times 10^{-6}\;{\mathrm{MPa}}^{-1}$$

where $V_{R0}=4909.39$ m/s is the stress-free surface-wave velocity for this frequency–radius combination (Table 2), and $L_{\mathrm{eff}}=60$ mm is the prescribed propagation distance.

**Figure 10:**
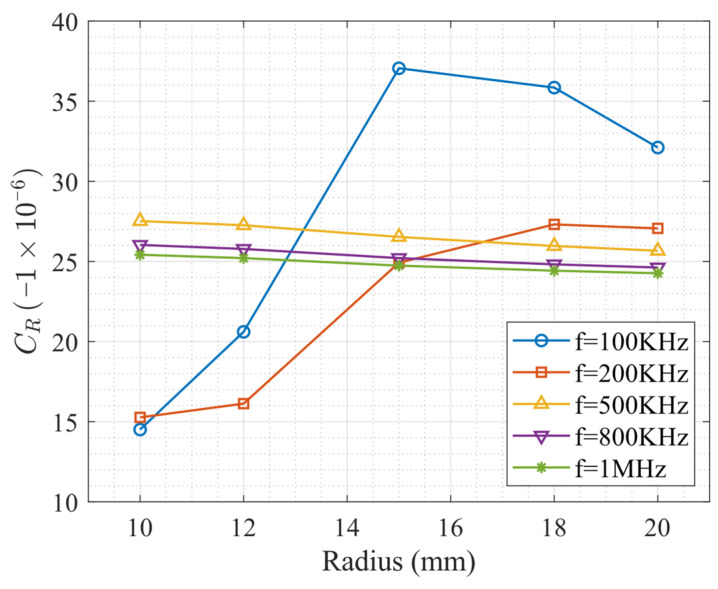
Acoustoelastic coefficient *C_R_* as a function of cylinder radius for different excitation frequencies.

These results are presented in Figure 10. Although the curves in Figure 10 exhibit trends similar to those observed in Figure 9, the transformation from ${\hat{C}}_{R}$ to $C_{R}$ is not linear, owing to the coupled dependence of the surface wave velocity on both excitation frequency and cylinder radius.

In this section, the acoustoelastic response of cylindrical surface waves under axial stress was quantified through finite element simulations. By introducing incremental prestress via modified material parameters, stress-induced time delays were extracted for a range of excitation frequencies and cylinder radii. The resulting acoustoelastic coefficients reveal a clear dependence on both geometric curvature and excitation frequency, with frequency effects becoming dominant in the higher-frequency regime. These results provide a calibrated description of the effective acoustoelastic behavior of surface waves on cylindrical structures and establish a quantitative basis for the discussion presented in the following section.

## 5. Conclusions

This study investigated the influence of surface curvature and excitation frequency on the acoustoelastic behavior of surface waves propagating along cylindrical structures. Motivated by the widespread practice of directly applying planar acoustoelastic formulations to curved components, a systematic numerical and theoretical framework was established to quantify the validity and limitations of this assumption.

Three-dimensional transient finite element simulations were performed for cylindrical specimens with varying radii and excitation frequencies, covering a wide range of wavelength-to-radius ratios. Surface-wave propagation, reception, and time-of-flight variations under incremental axial stress were analyzed in detail. By comparing stress-free and prestressed cases, curvature- and frequency-dependent acoustoelastic coefficients were extracted and evaluated.

The results demonstrate that both surface curvature and excitation frequency have a pronounced effect on the apparent acoustoelastic response. At low frequencies, where the wavelength is comparable to or larger than the cylinder radius, surface waves exhibit significant penetration into the cross-section, leading to strong curvature-induced deviations in wave velocity and acoustoelastic sensitivity. In contrast, at higher frequencies, the surface wave field becomes increasingly localized near the outer surface, and the influence of curvature diminishes, resulting in acoustoelastic coefficients that approach those predicted by planar theory. Furthermore, the combined dependence of surface wave velocity on frequency and radius leads to a nontrivial transformation between different definitions of the acoustoelastic coefficient.

These findings indicate that the direct application of planar acoustoelastic coefficients to curved structures may introduce non-negligible errors, particularly in low-frequency regimes or for components with small radii. The present results provide quantitative guidance for selecting appropriate excitation frequencies and interpreting surface wave measurements in cylindrical components. The proposed framework offers a practical reference for curvature-aware stress evaluation in surface-wave-based nondestructive testing and lays the groundwork for future experimental validation and model-based correction strategies.

It should be noted that the present study is based exclusively on three-dimensional transient finite element simulations. Although experimental validation is highly desirable, practical challenges currently limit its implementation. In particular, low-frequency surface-wave excitation requires transducers with relatively large dimensions, while the cylindrical specimens considered in this study have comparatively small radii. This mismatch between transducer size and specimen geometry makes controlled experimental measurements at low frequencies difficult to achieve with sufficient accuracy.

Despite these limitations, ongoing work is focused on developing suitable experimental configurations to validate the numerical findings presented herein. Future studies will aim to combine optimized transducer design with carefully selected specimen dimensions to enable experimental characterization of curvature-dependent acoustoelastic surface-wave behavior. Such efforts will further enhance the applicability of the proposed numerical framework to practical nondestructive stress evaluation.

## Figures and Tables

**Table 1:** Material properties of r 6061-T6 aluminum [34] and PMMA used in the finite element simulations.

Property	Aluminum 6061	PMMA
Density *ρ* (kg/m^3^)	2700	1210
Lamé Constant *λ* (GPa)	54.9	35.2
Lamé Constant *μ* (GPa)	26.5	23.5
Murnaghan Constant *l* (GPa)	−218.5	-
Murnaghan Constant *m* (GPa)	−339.0	-
Murnaghan Constant *n* (GPa)	−416.0	-

**Table 2:** Stress-free surface wave velocities for different excitation frequencies and cylinder radii. All velocities are given in m/s.

Frequency	Cylinder Radius (mm)				
	10	12	15	18	20
100 kHz	4909.39	3120.20	2896.62	2893.30	2893.91
200 kHz	4577.03	3054.36	2895.04	2893.47	2894.26
500 kHz	3833.04	2959.52	2893.36	2894.26	2895.24
800 kHz	3394.52	2924.00	2893.34	2895.06	2896.06
1 MHz	3224.79	2912.35	2893.59	2895.53	2896.51

**Table 3:** Wavelength-to-radius ratios *λ*/*R* corresponding to different excitation frequencies and cylinder radii, calculated using the stress-free surface wave velocities listed in Table 2.

Frequency	Cylinder Radius (mm)				
	10	12	15	18	20
100 kHz	49.09	26.00	19.31	16.07	14.47
200 kHz	22.89	12.73	9.65	8.04	7.24
500 kHz	7.67	4.93	3.86	3.22	2.90
800 kHz	4.24	3.05	2.41	2.01	1.81
1 MHz	3.22	2.43	1.93	1.61	1.45

## Data Availability

The original contributions presented in this study are included in the article. Further inquiries can be directed to the corresponding author.
